# Multi-layered NiO_y_/NbO_x_/NiO_y_ fast drift-free threshold switch with high I_on_/I_off_ ratio for selector application

**DOI:** 10.1038/s41598-017-04529-4

**Published:** 2017-06-22

**Authors:** Jaehyuk Park, Tobias Hadamek, Agham B. Posadas, Euijun Cha, Alexander A. Demkov, Hyunsang Hwang

**Affiliations:** 1 Department of Material Science and Engineering, Pohang University of Science and Technology, Pohang, 790-784 Korea; 2 Department of Physics, The University of Texas at Austin, Austin, Texas 78712 USA

## Abstract

NbO_2_ has the potential for a variety of electronic applications due to its electrically induced insulator-to-metal transition (IMT) characteristic. In this study, we find that the IMT behavior of NbO_2_ follows the field-induced nucleation by investigating the delay time dependency at various voltages and temperatures. Based on the investigation, we reveal that the origin of leakage current in NbO_x_ is partly due to insufficient Schottky barrier height originating from interface defects between the electrodes and NbO_x_ layer. The leakage current problem can be addressed by inserting thin NiO_y_ barrier layers. The NiO_y_ inserted NbO_x_ device is drift-free and exhibits high I_on_/I_off_ ratio (>5400), fast switching speed (<2 ns), and high operating temperature (>453 K) characteristics which are highly suitable to selector application for x-point memory arrays. We show that NbO_x_ device with NiO_x_ interlayers in series with resistive random access memory (ReRAM) device demonstrates improved readout margin (>2^9^ word lines) suitable for x-point memory array application.

## Introduction

The transition metal oxide NbO_2_ has gained significant interest over the years due to its wide range of applications such as optical sensors and various electronic devices due to its insulator-to-metal transition (IMT) characteristics^1–4^. Recently, several groups reported that the electrically-driven IMT characteristics of NbO_2_ as a selector device integrated with resistive switching random access memory (ReRAM) can suppress the leakage current of an x-point memory array^5–12^.

However, the I_on_/I_off_ ratio of such selectors is limited. Additionally, the intrinsic electrically-driven IMT mechanism of NbO_2_ has not yet been fully understood. The most widely used model to explain electrically-driven IMT mechanism of NbO_2_ is Joule-heating induced temperature driven transition model^8^. In this model, NbO_2_ can change to metallic state from insulating state at a certain voltage due to Joule-heating of filamentary activated metallic regions. However, the Joule-heating model conflicts with the fact that transition temperature of NbO_2_ (1080 K) is much higher than the temperature that can be induced by Joule-heating of the insulating state of NbO_2_^9^. A number of groups have reported that NbO_x_ can change its resistance far below the IMT temperature of NbO_2_ (1080 K) based on thermal runaway model with Poole-Frenkel simulation^13–15^. However, IMT mechanism of NbO_x_ under electrical field (E-field) is still not well understood. In this paper, we fabricated highly crystalline NbO_2_ film using molecular beam epitaxy (MBE) method and sputter-deposited NiO_y_ barrier inserted NbO_x_ device to analyze the underlying IMT mechanism of NbO_2_ film. Based on the electroforming analysis performed in previous research^16^, we examined the dependence of delay time on various applied voltages and temperatures to study the IMT mechanism of the film. Moreover, by comparing energy barriers for metallic phase nucleation with a calculated minimum energy pathway (MEP) value of the Peierls transition in NbO_2_ and with energy barriers for oxygen vacancy diffusion driven IMT in NbO_2_, we found that the IMT proceeds through a Peierls transition. We conclude that the IMT mechanism of NbO_2_ devices is consistent with a Peierls phase transition and follows the field induced nucleation theory. Although the IMT occurs within NbO_2_ by Peierls transition, we found that the leakage current of the insulating state is dominated by the interfacial defects between electrode and NbO_2_. Therefore, interface engineering was necessary to reduce the leakage current of NbO_2_ to improve its performance as a selector device. We revealed that insufficient Schottky barrier height between electrode and NbO_2_ formed as a result of interfacial defects, which increased the conductivity of insulating state.

Interface defects were successfully suppressed and a higher Schottky barrier can be formed by inserting a thin NiO_y_ barrier layer between electrode and sputtered-NbO_x_ (W/NiO_y_/NbO_x_/NiO_y_/W). As a result, the leakage current of W/NiO_y_/NbO_x_/NiO_y_/W device was significantly decreased and the device exhibited high I_on_/I_off_ ratio (>5400). The W/NiO_y_/NbO_x_/NiO_y_/W device exhibits very fast transition speed (<2 ns) and excellent operating thermal stability (>453 K). The W/NiO_y_/NbO_x_/NiO_y_/W device can have very fast delay time (<30 ns) and is drift-free, which are highly suitable attributes for selector application in x-point memory array.

We demonstrated the 1S1R (1 selector – 1 ReRAM) unit cell and showed improved readout margin (>2^9^ word lines) by using W/NiO_y_/NbO_x_/NiO_y_/W device.

## Result and Discussions

**Figure 1:**
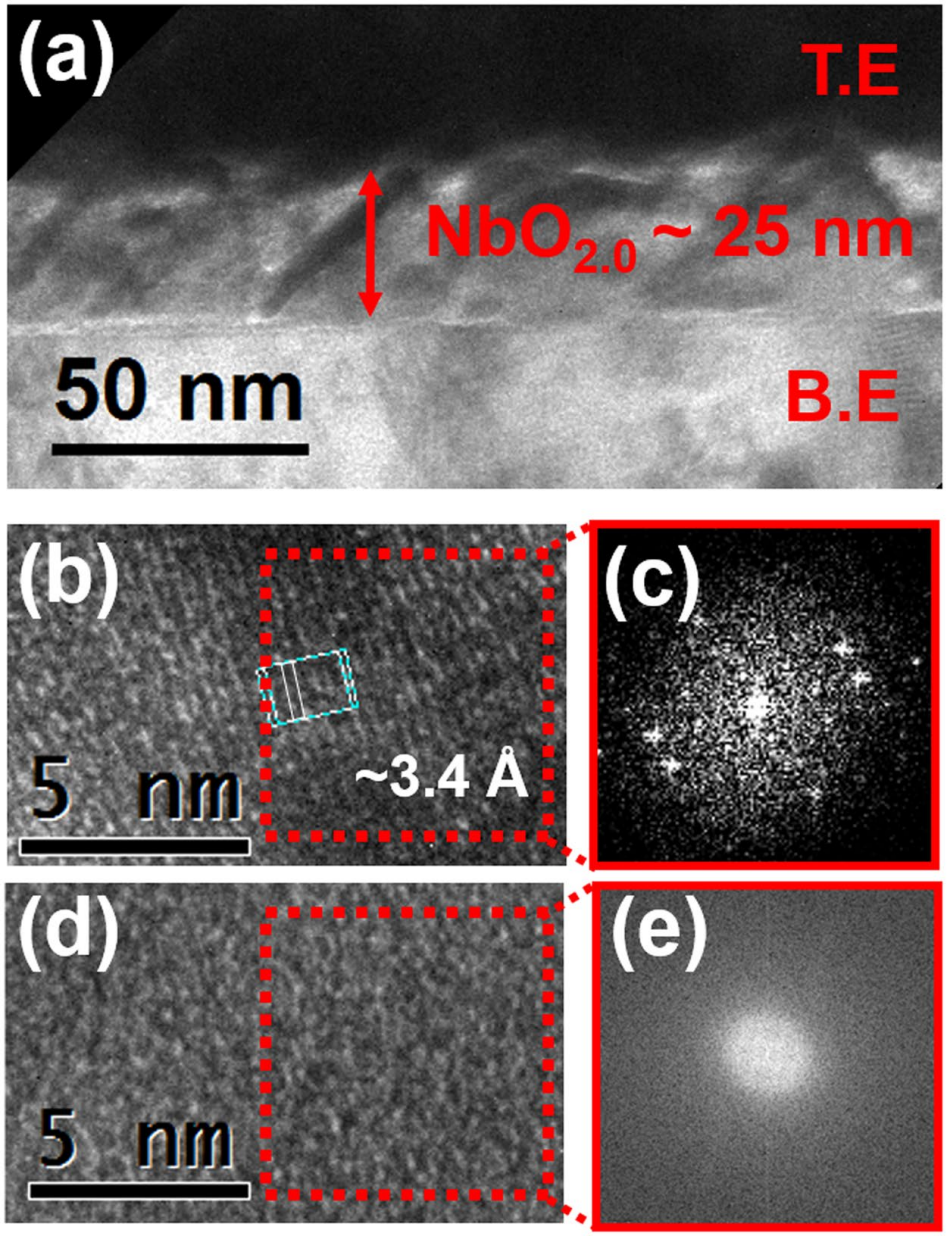
(**a**) Overall TEM image of device structure of MBE deposited NbO_2_ film. Thickness was about 25 nm. (**b**) HRTEM image and (**c**) FFT image of MBE deposited NbO_2_ film which has poly-crystalline state. The lattice constant was about 3.4 Å and it was corresponding with (400) direction of NbO_2_. (**d**) HRTEM image and (**e**) FFT image of sputter deposited NbO_x_ film shows that it has purely amorphous state.

The cross-sectional transmission electron microscopy (TEM) image shows film structure and crystalline state of both MBE and sputter deposited films (Fig. 1). The films were analyzed by *in-situ* X-ray photoelectron spectroscopy (XPS) to confirm that the correct phase and composition were achieved. Details of the MBE growth and XPS phase identification are found in ref. 17 and XPS results for the sputtered films are shown in Supplementary Fig. S1 ^18, 19^. The MBE NbO_2_ film was polycrystalline with a lattice constant of 3.4 Å. This corresponds to the d-spacing of the (400) planes of the insulating body-centered tetragonal NbO_2_ phase^20^. On the other hand, the sputter-deposited NbO_x_ film was amorphous as-grown.

In comparison with the sputter-deposited NbO_x_ film, the electroforming process was mostly eliminated in the MBE-deposited NbO_2_ film, as shown in Supplementary Fig. S2. In the case of the sputter-deposited film, the pristine state of the film was amorphous. Therefore, electroforming was needed to form crystalline tetragonal NbO_2_ regions within the amorphous matrix to exhibit IMT^16^. On the other hand, electroforming was not needed in the case of MBE-deposited NbO_2_ film because the pristine state of the film was already polycrystalline tetragonal NbO_2_. The difference in electroforming and IMT process between sputter- and MBE-deposited films is summarized in Supplementary Fig. S3. Because there is no longer any need for electroforming, the IMT process in MBE-deposited NbO_2_ films can be precisely analyzed.

The mechanism of IMT of NbO_2_ under E-field was widely interpreted by Joule-heating model, and this model suggests that electrically induced Joule-heating generate the sufficient heat over IMT temperature of NbO_2_ (1080 K)^8, 10^. However, the IMT temperature of NbO_2_ (1080 K) is much higher than the temperature that can achieved by Joule-heating within the insulating state of NbO_2_^9^. Therefore, several researches proposed that the mechanism of IMT under E-field is the result of thermal runaway model^13–15^. These researches simulated the conductivity of NbO_2_ device as a function of temperature and E-field by fitting the I-V characteristics with Poole-Frenkel model. They showed that IMT can take place far below IMT temperature of NbO_2_ (1080 K) by thermal runaway, which successfully resolved the main drawback of classical Joule-heating IMT model.

We take a different perspective by using field-induced nucleation theory to explain IMT mechanism in this research. Devices that abruptly change their resistance at a certain electric field, such as phase change random access memory (PRAM) or VO_2_-based IMT devices, energetically favor metallic nuclei with a cylindrical shape upon nucleation via the applied electric field^21–25^. Similarly, the field-induced IMT of NbO_2_ is expected to result of a Peierls transition of conductive NbO_2_ (metallic, i.e. rutile NbO_2_) regions formed as cylindrical shape nuclei within an insulating NbO_2_ matrix (tetragonal, distorted rutile NbO_2_) under the influence of an electric field^21, 22^. The formation of nuclei is favorable and forms a conductive path through the insulating host material. The free energy the system ∆G consist of:

1 $${\rm{\Delta }}{\rm{G}}={\rm{A}}{\rm{\sigma }}-{\rm{\Omega }}{\rm{\mu }}+{{\rm{W}}}_{{\rm{E}}}$$

**Figure 2:**
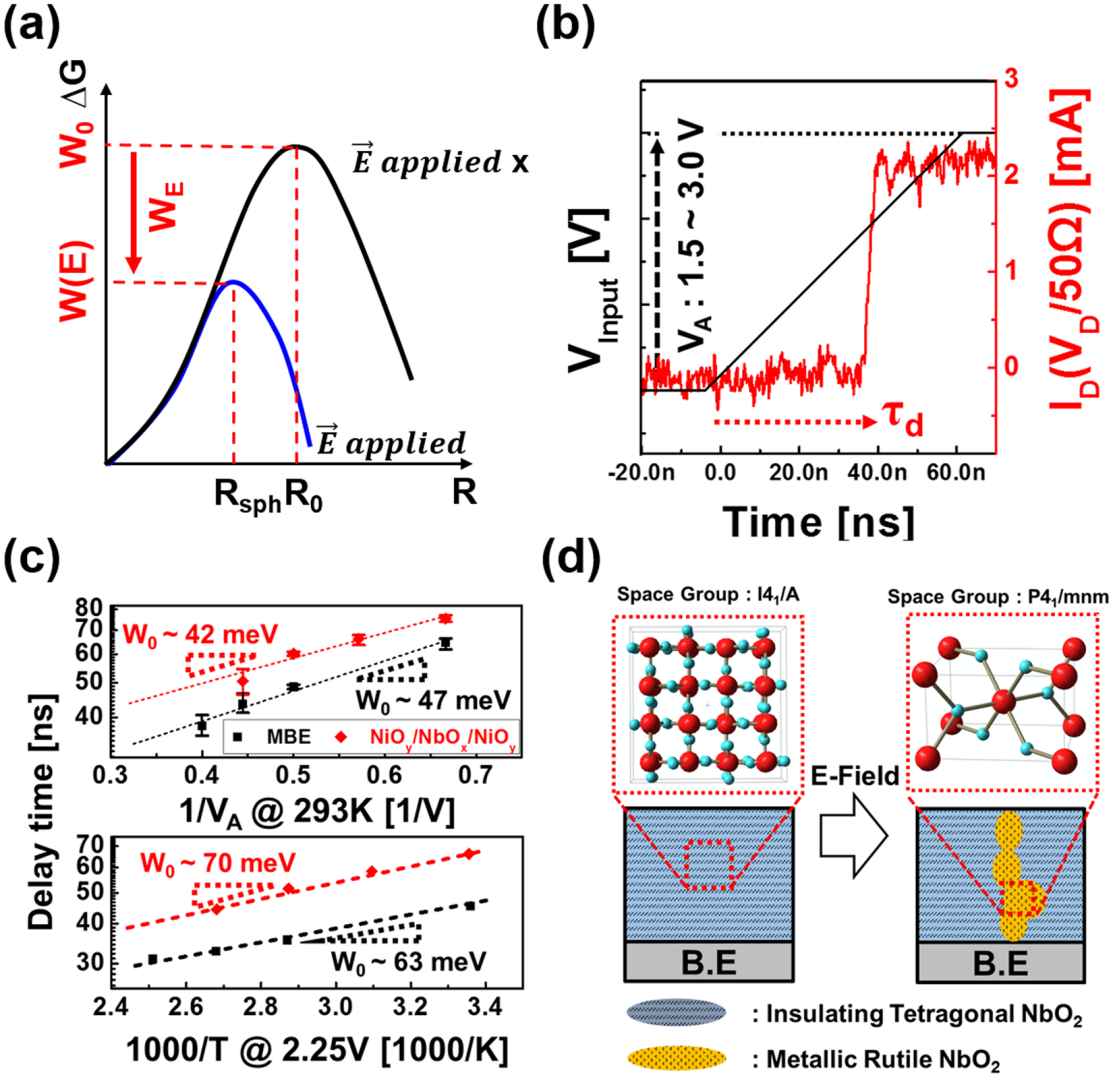
(**a**) Energy barrier W_0_ to nucleate without E-field can be lowered by E-field. W(E) is effective barrier energy to nucleate with E-field. (**b**) Measurement example to detect the delay time predicted by the nucleation theory during IMT. (**c**) Arrhenius’s plot of delay time versus voltage and temperature of NbO_2_ film. (**d**) The schematic diagram of phase transition: the transition is the result of Pielers phase transition.

Here, σ and μ are the surface tension and the chemical potential difference between the two NbO_2_ phases, respectively. The transition energy barrier is lowered by an external electric field (${{\rm{W}}}_{{\rm{E}}}=\,-\frac{\varepsilon {E}^{2}{\rm{\Omega }}}{8n\pi }$, where *ε* is the dielectric constant of the host and n is the depolarizing factor ($n=\frac{1}{3}$ for a sphere)) as shown in Fig. 2(a).

If we assume that spherical nuclei exist in zero-field (W_E_ = 0), then the surface area and volume of the nuclei can be defined as A = 4πR^2^ and Ω = 4πR^3^/3, respectively. By using the differential form of the free energy at zero-field (${\rm{\Delta }}{\rm{G}}=4{{\rm{\pi }}{\rm{R}}}^{2}{\rm{\sigma }}-4{{\rm{\pi }}{\rm{R}}}^{3}{\rm{\mu }}/3$), we can define the energy barrier at zero-field (${{\rm{W}}}_{0}=16\pi {{\rm{\sigma }}}^{3}/3{{\rm{\mu }}}^{2}$) and the equivalent radius of the nuclei (R_0_ = 2σ/μ). However, because the nuclei with cylindrical shape are energetically more favorable than spherical ones when an E-field is applied^22^, it is preferable to modify Equation (1) as follows:

2 $${\rm{\Delta }}{\rm{G}}=\frac{{{\rm{W}}}_{0}}{2}(\frac{3Rh}{{R}_{0}^{2}}-\frac{3{R}^{2}h}{{R}_{0}^{3}}-\frac{{E}^{2}{h}^{3}}{{E}_{0}^{2}{R}_{0}^{3}}),$$

with R being the cylinder radius and ***h*** its height. Following from Eq. (2), the reduced barrier energy W(E) under E-field is given by:

3 $${\rm{W}}({\rm{E}})={{\rm{W}}}_{0}{\alpha }^{3/2}{E}_{0}/E\,={W}_{0}{\alpha }^{3/2}d{E}_{0}/V.$$

Here, E_0_ is the voltage acceleration factor of the first order, independent of the external voltage or temperature, and its conventional value is 1 MV/cm^22^, d is the thickness of film; and α is geometric factor of cylinder radius compared with equivalent radius of the nuclei at zero-field (R = αR_0_) where 0.1 ≤ α < 0.5. We assume α = 0.5 because this value corresponds to the maximum barrier^23^.

The theory predicts the delay time between application of the field and the switching event expressed as^24^:

4 $${{\rm{\tau }}}_{{\rm{d}}}={{\rm{\tau }}}_{0}\exp (-\frac{W(E)}{kT})={{\rm{\tau }}}_{0}\exp (-\frac{{W}_{0}{\alpha }^{3/2}{E}_{0}d}{kTV})$$

The value of τ_d_ for the film was measured by using rising ramp pulses that can minimize RC delay effect and can reveal the delay time with various voltages (V_A_) and temperatures^26^. τ_d_ is defined as the point where I_D_ (V_D_/50 Ω) suddenly increases, as shown in Fig. 2(b). Here, τ_d_ decreases exponentially with V_A_ and temperature. Figure 2(c) shows that the relation between temperature/V_A_ and τ_d_ can be described by an Arrhenius plot, which follows Equation (4).

We found that the experimentally determined value of the zero field barrier W_0_, which is 47–63 meV, agrees well with the calculated minimum energy pathway (MEP) found between rutile and tetragonal NbO_2_ during the Peierls transition, which is 43 meV^27^. The alternative mechanism of diffusion or electromigration of oxygen (vacancies) has also been discussed in terms of the IMT in niobium oxides^11, 28, 29^. The values of the diffusion barrier height of roughly 290–550 meV deduced from the diffusion studies in Nb_2_O_5_ reported in ref. 30. Also, the observed oxygen diffusion coefficient in NbO_2_ is lower than that of the pentoxide (indicating a higher diffusion barrier for NbO_2_ than for Nb_2_O_5_). Therefore, we can conclude that oxygen diffusion is energetically less favorable than Peierls phase transition due to the high barrier for oxygen diffusion. Furthermore, the diffusion barrier at zero field is estimated to be reduced by only ~10 meV under electric field application considering the electric potential drop along a typical diffusion length of ~1 Å. Therefore, IMT of NbO_2_ is likely due to a Peierls phase transition through field induced nucleation rather than oxygen electromigration. The events that occur during IMT are illustrated in Fig. 2(d).

The expected transition speed of NbO_2_ film is quite fast because only short-range atomic arrangement is needed for the transition (Peierls transition). Therefore, NbO_2_ based IMT device is well suited for selector device in x-point memory array. However, sufficiently high resistivity in the insulating state of NbO_x_ film has not yet been obtained. Moreover, the off-current of NbO_2_ film can be suppressed under 1 nA at 1 V (Area = 50 × 50 nm^2^, Thickness = 25 nm) because insulating state of NbO_2_ conductivity is about 10^−4^ S/cm^31^. Likely, the relatively high conductivity of the insulating state of NbO_x_ originates from interface defects between electrode and NbO_x_ layer. In fact, many defects (Grain boundary, dislocation, and point defects) are observed between electrode and MBE-deposited NbO_2_ film by TEM image (Supplementary Fig. S4). Additionally, these interface defects were observed in sputter-deposited NbO_x_ device from our previous research^32^.

These defects can pin the Fermi level between electrode and NbO_x_ layer. As a result, the device does not have a sufficiently high Schottky barrier. These defects can be eliminated and a high Schottky barrier can be obtained by inserting a NiO_y_ layer, which consists of NiO and Ni_2_O_3_ phases (Supplementary Fig. S1), between electrode and NbO_x_ layer (W/NiO_y_/NbO_x_/NiO_y_/W) as shown in Supplementary Fig. S5 ^33^. Based on DC I-V characteristics at various temperatures for both devices, current-temperature dependencies at low field (V = 0.1 V, saturation region) for both devices follow the Richardson relation (Eq. (5)) and the effective Schottky barrier can be obtained using the slope of the Richardson plot (Supplementary Fig. S5). W/NiO_y_/NbO_x_/NiO_y_/W devices have higher Schottky barrier height (φ_B_ ~ 0.25 eV) than W/NbO_x_/W devices (φ_B_ ~ 0.15 eV).

5 $${{\rm{J}}}_{0} \sim \,{A}^{\ast }{T}^{2}\exp (-\frac{q{\phi }_{Bp}}{kT})$$

(J_0_ = Current density at saturation region, A* = Richardson constant, T = temperature, k = Boltzmann constant, q = electronic charge, φ_Bp_ = effective schottky barrier energy).

Before comparing the device performance of W/NiO_y_/NbO_x_/NiO_y_/W device with W/NbO_x_/W device, we analyzed the delay time of the W/NiO_y_/NbO_x_/NiO_y_/W. Interestingly, the zero field barrier W_0_ of W/NiO_y_/NbO_x_/NiO_y_/W device (42–70 meV), which was extracted from delay time Arrhenius plot, also corresponds well to the calculated minimum energy pathway (MEP) found between rutile and tetragonal NbO_2_ during the Peierls transition, which is 43 meV. This value is the same as that obtained from MBE film analysis (Fig. 2(c)). These results inferred that the barrier layers were not affected by transition mechanism of NbO_x_. Meanwhile, interface structure of NbO_x_ device can control the conductivity of insulating state of the device. Therefore, we can suppress the high conductivity of NbO_x_ film by simply inserting NiO_y_ barrier layer without compromising fast transition characteristics of NbO_x_ IMT layer.

**Figure 3:**
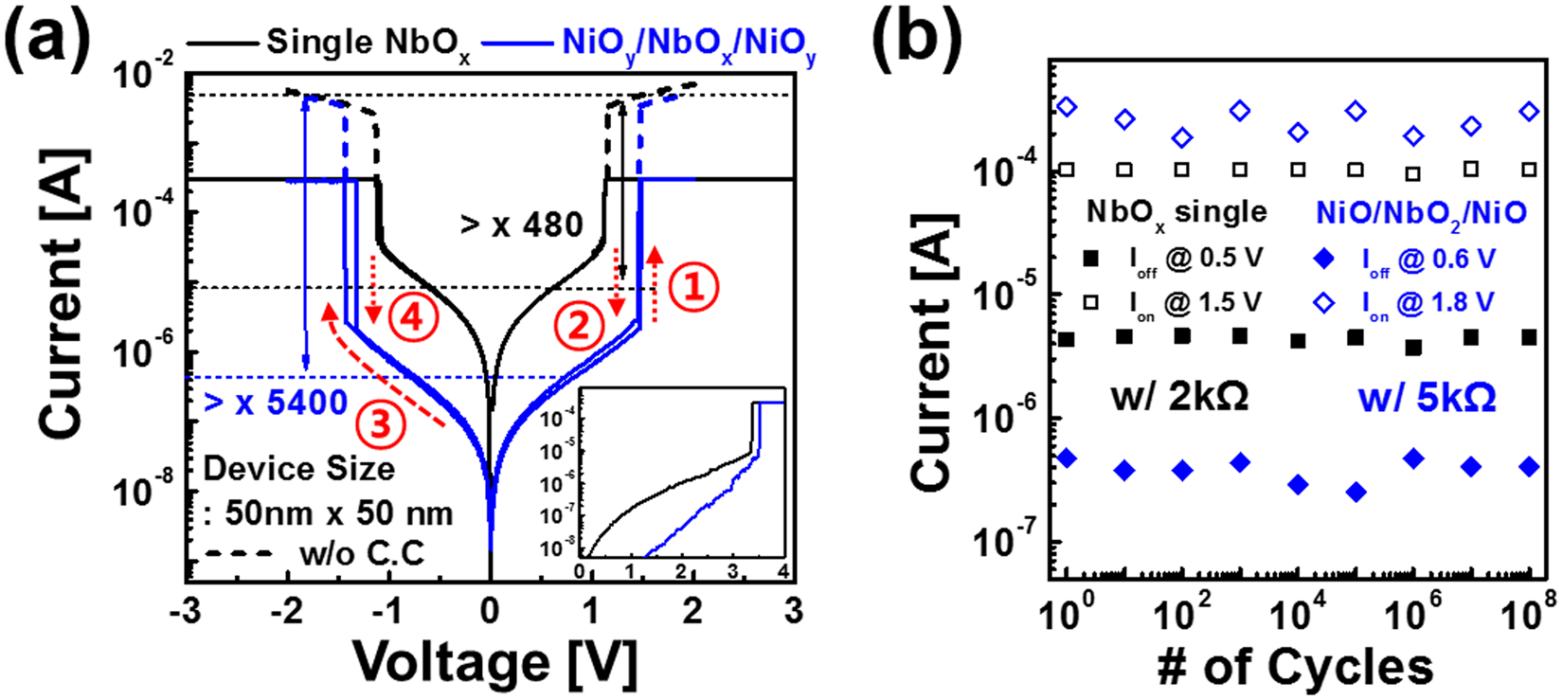
(**a**) DC I-V characteristics of NbO_x_ single layer device and NiO_y_/NbO_x_/NiO_y_ device during threshold switching after forming process (inset) and (**b**) AC endurance of both devices.

Figure 3(a) shows IMT characteristics after electroforming both W/NbO_x_/W and W/NiO_y_/NbO_x_/NiO_y_/W devices. Compared with the W/NbO_x_/W device, the W/NiO_y_/NbO_x_/NiO_y_/W device exhibited decreased conductivity in the insulating state. The I_on_/I_off_ ratio of W/NiO_y_/NbO_x_/NiO_y_/W device improved to >5400 from >480, which is the I_on_/I_off_ ratio of the W/NbO_x_/W device. Both devices have superior endurance that persisted over 10^8^ AC cycles as shown in Fig. 3(b). The W/NiO_y_/NbO_x_/NiO_y_/W device has very uniform device-to-device and cycle-to-cycle stability during several DC I-V sweeps (Supplementary Fig. S6).

Moreover, we measured the transition time and delay time of W/NiO_y_/NbO_x_/NiO_y_/W device to investigate the temporal characteristics of the device. The device has a transition time under 2 ns and a delay time down to 30 ns for variable voltage ramps (Supplementary Fig. S7). We expect that the delay time of W/NiO_y_/NbO_x_/NiO_y_/W can be even shorter for square pulses.

**Figure 4:**
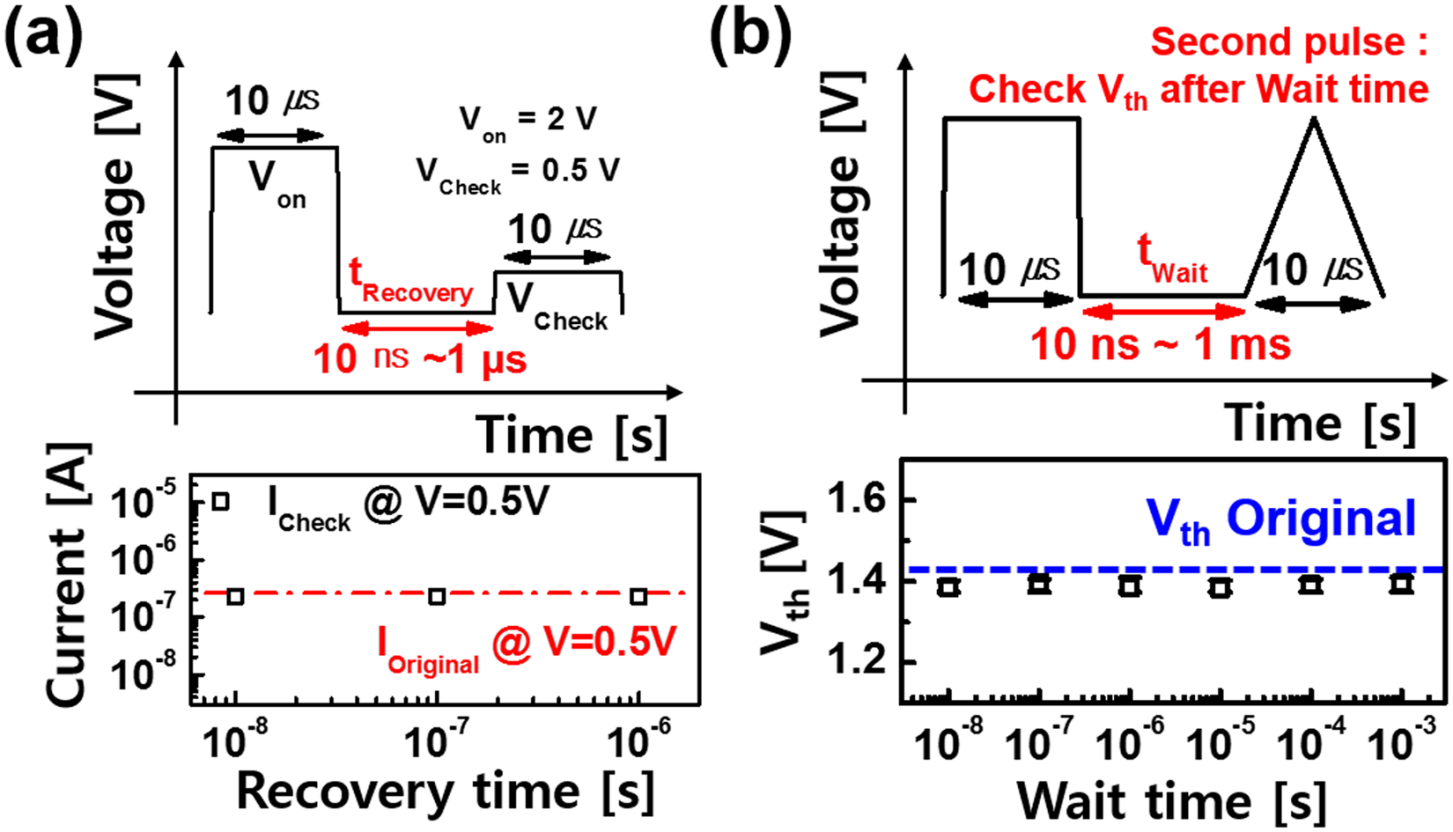
(**a**) NiO_y_/NbO_x_/NiO_y_ device can recover its insulating state under 10 ns and (**b**) maintain its TS characteristics within different wait time (drift-free characteristics).

Since IMT mechanism of NbO_x_ is a second-order structural transition of the Peierls type and involves only very short range atomic displacements, the drift-free characteristic is available in NbO_x_ based device. As a matter of fact, Fig. 4(a) shows that W/NiO_y_/NbO_x_/NiO_y_/W device can recover its insulating state under less than 10 ns. Figure 4(b) illustrates the drift-free operation of the W/NiO_y_/NbO_x_/NiO_y_/W device when V_th_ does not change at different time intervals^34^. These results indicate that the W/NiO_y_/NbO_x_/NiO_y_/W device can be used for fast operating applications.

**Figure 5:**
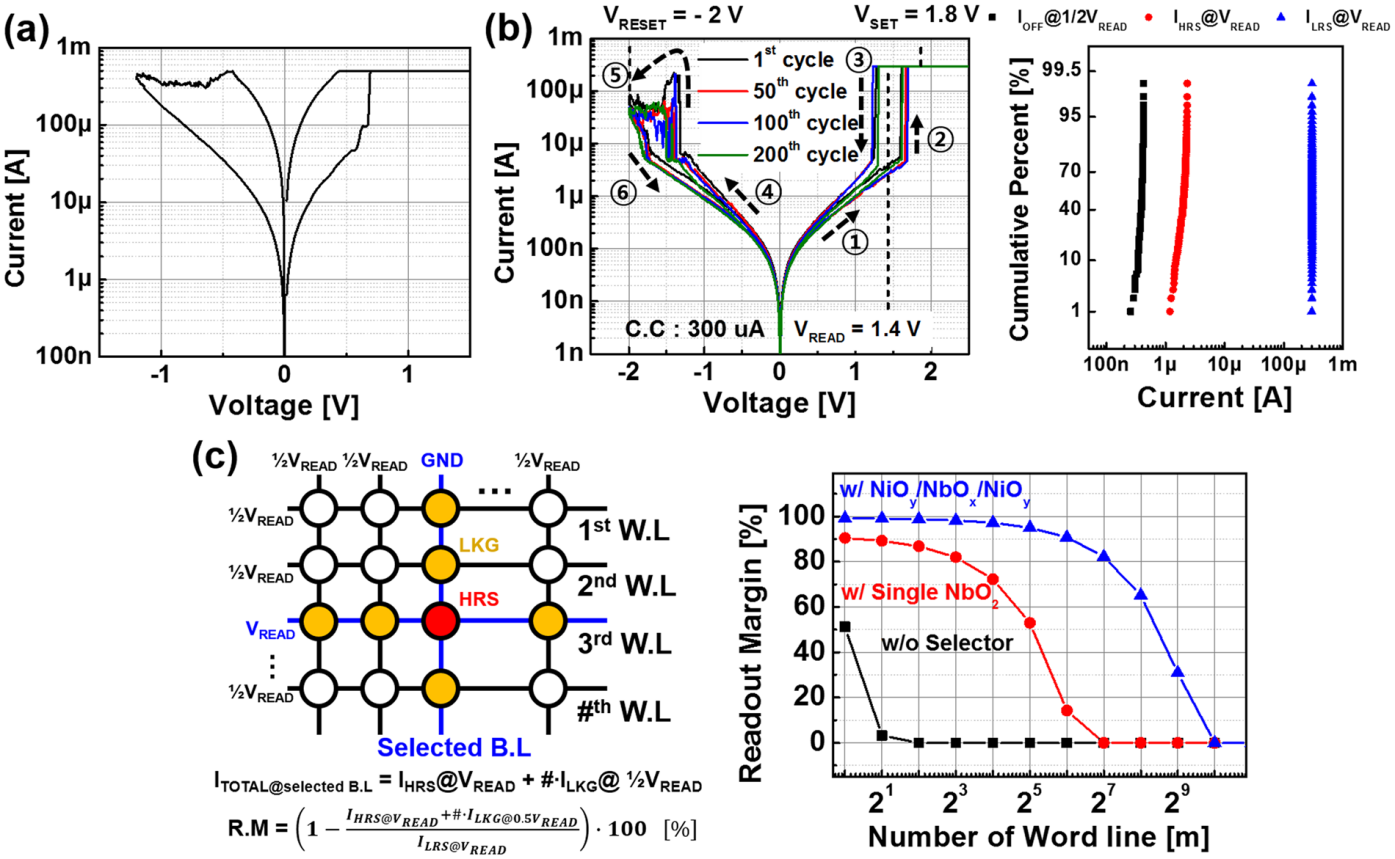
(**a**) DC I-V characteristic of TiN/Ti/HfO_x_/TiN ReRAM and (**b**) DC I-V characteristic of NiO_y_/NbO_x_/NiO_y_ selector device with TiN/Ti/HfO_x_/TiN ReRAM. (**c**) Readout margin of TiN/Ti/HfO_x_/TiN ReRAM with single NbO_x_ and NiO_y_/NbO_x_/NiO_y_ selectors.

We also evaluated the feasibility of x-point memory array using a novel W/NiO_y_/NbO_x_/NiO_y_/W device. The W/NiO_y_/NbO_x_/NiO_y_/W device was connected in series with a TiN/Ti/HfO_x_/TiN ReRAM device (ReRAM, 1 R) which has DC I-V characteristic shown in Fig. 5(a). Set voltage (V_set_) and reset voltage (V_reset_) of ReRAM is about 0.6 V and −1.2 V, respectively. To prevent the hard breakdown of 1 R, we set the compliance current to 500 μA during operation. Figure 5(b) shows DC I-V characteristics of 1S-1R device with superior DC endurance (>300 cycles). The V_set_ and V_reset_ of 1S-1R was about 1.8 V and −2 V, respectively. The state of the device is determined by applying a read voltage (V_read_) of 1.4 V.

We simulated the x-point memory using novel W/NiO_y_/NbO_x_/NiO_y_/W selector based on measurement in Fig. 5(b). Since the leakage current of unselected cell at ½V_read_ is suppressed to about 300 nA in both LRS and HRS state by adopting the W/NiO_y_/NbO_x_/NiO_y_/W selector, we demonstrate that the readout margin (Eq. (6)) can improve up to 2^9^ vs. 2^1^ word lines (W.L.) as shown in Fig. 5(d) ^35, 36^.

6 $${\rm{R}}.{\rm{M}}=(1-\frac{{I}_{HRS@{V}_{READ}}+\#\cdot {I}_{LKG@0.5{V}_{READ}}}{{I}_{LRS@{V}_{READ}}})\cdot 100\,[ \% ]$$

(# = Number of word line(s))

## Conclusion

We have successfully fabricated NbO_2_ poly-crystalline film using MBE, which does not require an electroforming process. We find that the IMT in NbO_2_ undergoes the Peierls phase transition through the field-induced nucleation with the formation of a conductive filament of rutile NbO_2_ in an insulating host matrix of tetragonal NbO_2_. We also showed that the leakage current of NbO_2_ IMT device originates from the insufficient Schottky barrier height between electrode and NbO_x_ layer as a result of interfacial defects. Sufficiently high Schottky barrier and improved IMT characteristics can be obtained by introducing a NiO_y_ layer between electrode and NbO_x_ layer. A novel W/NiO_y_/NbO_x_/NiO_y_/W device has high I_on_/I_off_ ratio (>5400), high operating temperature (>453 K), fast transition speed (<2 ns) and drift-free operation. We employed the W/NiO_y_/NbO_x_/NiO_y_/W device as a selector device on ReRAM memory cell. Due to the excellent selector characteristics of W/NiO_y_/NbO_x_/NiO_y_/W device, we show a significantly improved readout margin (up to 2^9^ word lines) is possible in a large x-point memory array.

## Methods

First, to analyze the IMT mechanism under E-field, we fabricated NbO_x_ films using both MBE and RF-sputtering. About 25 nm-thick NbO_x_ film was deposited by MBE and RF-sputtering on a 50 × 50 nm^2^ TiN bottom electrode (B.E). The MBE-deposited NbO_2_ film was deposited at 700 °C. Nb metal was evaporated from an electron beam source and molecular oxygen at a pressure of 5 × 10^−6^ Torr were used. The sputter-deposited NbO_x_ film was deposited at room temperature by RF reactive sputtering with a process gas of Ar/O_2_ (30 sccm/1.3 sccm), at a working pressure of 5 × 10^−3^ Torr and forward power of 100 W using a 2-inch Nb metal target. After both of NbO_x_ were deposited, positive photoresist was spincoated at 3000 rpm for 35 s and baked at 100 °C for 90 s. The photoresist were exposed under the lithography mask which has 50 × 50 um^2^ pattern and removed with developer to deposit contactable top electrode. Afterwards, W top electrode was deposited by RF reactive sputtering at room temperature with a process gas of Ar (30 sccm), at a working pressure of 5 × 10^−3^ Torr and forward power of 100 W using a 2-inch W metal target.

Secondly, to reduce the leakage current of NbO_x_ film, NiO_y_ barrier layer inserted NbO_x_ structure deposited on a 50 × 50 nm^2^ W bottom electrode (B.E). About 2-3 nm thick NiO_y_ layer was sputter deposited additionally by RF reactive sputtering with a process gas of Ar/O_2_ (30 sccm/2.0 sccm), at a working pressure of 5 × 10^−3^ Torr and forward power of 30 W using a 2-inch Ni metal target as a barrier layer between W electrodes and sputtered NbO_x_ layer. The condition for sputtering NbO_x_ is same with above. As a result, W/NiO_y_/NbO_x_/NiO_y_/W device was fabricated and its electrical characteristics compared to a W/NbO_x_/W control sample.

## Electronic supplementary material

Supplementary Information

## References

[1] Arshak, K., Hickey, G., Harris, J. & Ford, E. Ozone sensing properties of NbO_2_ thin films for health and safety applications. Sensors Applications Symp. 187–192 (2008).

[2] Shin SH, Halpern T, Raccah PM (1977). High-speed high-current field switching of NbO_2_. J. Appl. Phys..

[3] Philipp HR, Levinson LM (1979). NbO_2_ devices for subnanosecond transient protection. J. Appl. Phys..

[4] Lee JC, Durand WW (1984). Electrically stimulated optical switching of NbO_2_ thin films. J. Appl. Phys..

[5] Cha, E. *et al*. Nanoscale (~10nm) 3D vertical ReRAM and NbO_2_ threshold selector with TiN electrode. In IEEE IEDM Tech. Dig. 10.5.1–10.5.4 (2013).

[6] Kim, S. *et al*. Ultrathin (<10nm) Nb2O5/NbO2 hybrid memory with both memory and selector characteristics for high density 3D vertically stackable RRAM applications. Symp. on VLSI Tech. Dig. 155–156 (2012).

[7] Liu X (2011). Diode-less bilayer oxide (WO_x_–NbO_x_) device for cross-point resistive memory applications. Nanotechnology.

[8] Pickett MD, Williams RS (2012). Sub-100 fJ and sub-nanosecond thermally driven threshold switching in niobium oxide crosspoint nanodevices. Nanotechnology.

[9] Liu X (2012). Co-Occurrence of Threshold Switching and Memory Switching in Pt/NbO*x*/Pt Cells for Crosspoint Memory Applications. IEEE Elec. Dev. Lett..

[10] Liu X (2014). Reduced Threshold Current in NbO_2_ Selector by Engineering Device Structure. IEEE Elec. Dev. Lett..

[11] Nandi SK, Liu X, Venkatachalam DK, Elliman RG (2015). Threshold current reduction for the metal–insulator transition in NbO_2− x_-selector devices: the effect of ReRAM integration. J. Phys. D..

[12] Kang M, Yu S, Son J (2015). Voltage-induced insulator-to-metal transition of hydrogen-treated NbO_2_ thin films. J. Phys. D: appl. Phys..

[13] Funck C (2016). Multidimensional Simulation of Threshold Switching in NbO_2_ Based on an Electric Field Triggered Thermal Runaway Model. Adv. Elec. Mat..

[14] Slesazeck S (2015). Physical Model of Threshold Switching in NbO_2_ Based Memristors. RSC Adv..

[15] Gibson GA (2016). An accurate locally active memristor model for S-type negative differential resistance in NbO_x_. Appl. Phys. Lett..

[16] Park J, Cha E, Karpov IV, Hwang H (2016). Dynamics of electroforming and electrically driven insulator-metal transition in NbO_x_ selector. Appl. Phys. Lett..

[17] O’Hara A, Nunley TN, Posadas AB, Zollner S, Demkov AA (2014). Electronic and optical properties of NbO_2_. J. Appl. Phys..

[18] Bharti DC, Rhee SW (2013). Dielectric properties and X-ray photoelectron spectroscopic studies of niobium oxide thin films prepared by direct liquid injection chemical vapor deposition method. Thin Solid Films.

[19] Chen YS (2012). Microscopic mechanism for unipolar resistive switching behavior of nickel oxides. J. Phys. D..

[20] Magneli A, Andersson G, Sundkvist G (1955). Note on the crystal structure of niobium dioxide. Acta Chem. Scand..

[21] Chakraverty BK (1970). Metal-insulator transition; nucleation of a conducting phase in amorphous semiconductors. J. Non-Crystalline Solids.

[22] Pevtsov AB (2012). Evidence of field-induced nucleation switching in opal: VO_2_ composites and VO_2_ films. Phys. Rev. B..

[23] Karpov IV (2008). Evidence of field induced nucleation in phase change memory. Appl. Phys. Lett..

[24] Karpov VG, Kryukov YA, Karpov IV, Mitra M (2008). Field-induced nucleation in phase change memory. Phys. Rev. B..

[25] Madan H, Jerry M, Pogrebnyakov A, Mayer T, Datta S (2015). Quantitative Mapping of Phase Coexistence in Mott-Peierls Insulator during Electronic and Thermally Driven Phase Transition. ACS nano.

[26] Kim B (2007). Temperature dependence of Mott transition in VO_2_ and programmable critical temperature sensor. Appl. Phys. Lett..

[27] O’Hara A, Demkov AA (2015). Nature of the metal-insulator transition in NbO_2_. Phys. Rev. B..

[28] Hanzig F (2015). Effect of the stoichiometry of niobium oxide on the resistive switching of Nb_2_O_5_ based metal–insulator–metal stacks. J. Electron Spectros. Relat. Phenomena.

[29] Wang Y, Comes RB, Wolf SA, Lu J (2016). Threshold Switching Characteristics of Nb/NbO_2_/TiN Vertical Devices. J. Electron Devices Soc..

[30] Oechsner H, Giber J, Füßer HJ, Darlinski A (1985). Phase transition and oxide dissolution processes in vacuum-annealed anodic Nb_2_O_5_/Nb systems. Thin Solid Films.

[31] Janninck RF, Whitmore DH (1966). Electrical conductivity and thermoelectric power of niobium dioxide. J. Phys. Chem. Solids.

[32] Park J (2015). Improved threshold switching characteristics of multi-layer NbOx for 3-D selector application. Microelectronic Engineering.

[33] Islam R, Shine G, Saraswat KC (2014). Schottky barrier height reduction for holes by Fermi level depinning using metal/nickel oxide/silicon contacts. Applied. Phys. Lett..

[34] Karpov IV, Mitra M, Kau D, Spadini G (2007). Fundamental drift of parameters in chalcogenide phase change memory. J. Appl. Phys..

[35] Linn E, Rosezin R, Kügeler C, Waser R (2010). Complementary resistive switches for passive nanocrossbar memories. Nat. Mater..

[36] Koo, Y., Baek, K. & Hwang, H. Te-Based Amorphous Binary OTS Device with Excellent Selector Characteristics for X-Point Memory Applications. Symp. on VLSI Tech. Dig. 1–2 (2016).

