# Supplementary material for: Multi-layered NiOy/NbOx/NiOy fast drift-free threshold switch with high Ion/Ioff ratio for selector application
Source: Sci Rep. 2017 Jun 22;7:4068. doi: 10.1038/s41598-017-04529-4 (PMC5481432; doi:10.1038/s41598-017-04529-4)
Supplement: Supplementary file 1 — Supplementary Information [file 41598_2017_4529_MOESM1_ESM.doc]

**Supplementary Information**

**Multi-layered NiO_y_/NbO_x_/NiO_y_ fast drift-free threshold switch with high on/off ratio for selector application**

**Jaehyuk Park^1^, Tobias Hadamek^2^, Agham B. Posadas^2^, Euijun Cha^1^,**

**Alexander A. Demkov^2^ and Hyunsang Hwang^1*^**

^1^Department of Material Science and Engineering, Pohang University of Science and Technology, Pohang 790-784, Korea

^2^Department of Physics, The University of Texas at Austin, Austin, Texas 78712, USA

Correspondence and requests for materials should be addressed to H. Hwang (e-mail: hwanghs@postech.ac.kr)

**Supplementary Figure S1.** X-ray photoelectron spectroscopy (XPS) for sputtered NbO_x_ layer.


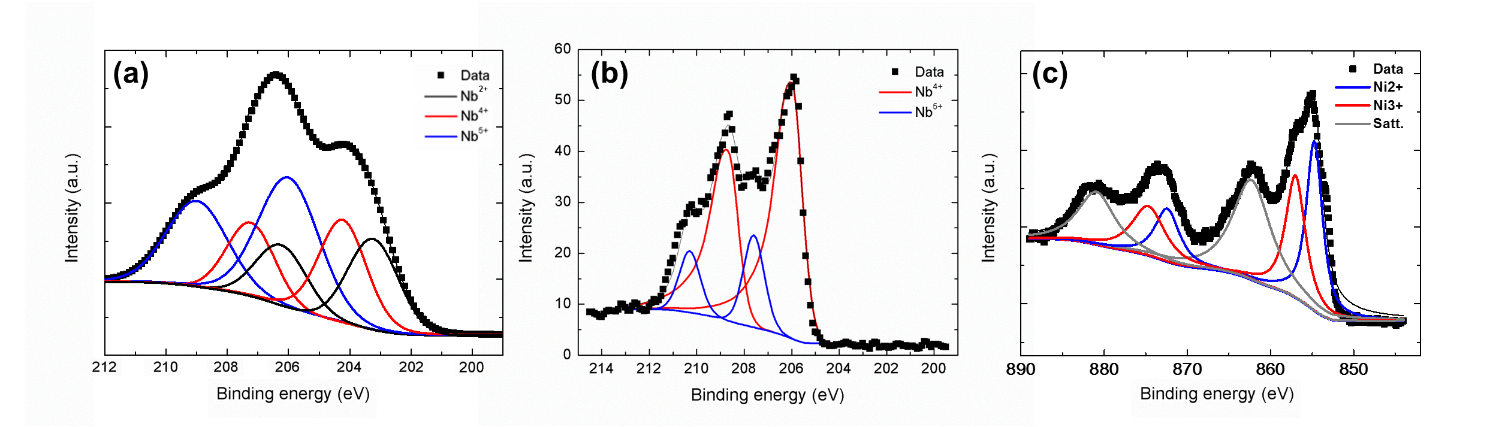
The XPS analysis result at supplementary Fig. S1 shows that (a) sputtered NbO_x_ layer consist of NbO, NbO_2_, and Nb_2_O_5_ phases, regardless of their crystalline states. The area (or peak) ratio of 3d_3/2_ and 3d_5/2_ peaks is 2:3, because each peak attributes to a (2j + 1) splitting pattern. The 3d_5/2_ binding energies of the NbO, NbO_2_, and Nb_2_O_5_ phases are 203.6 eV, 205 eV, and 206.4 eV, respectively [12,35]. The actual peaks for NbO_x_, corresponding to the 3d orbital, are shifted about 0.3–0.8 eV, because of sub-oxide phases such as Nb_2_O_3_ and Nb_3_O_7_. (b) The MBE-deposited NbO_2_ layer consist of NbO_2_ according to O’Hara, A.[17]. Nb_2_O_5_ detection was expected that result of surface oxidation by air exposure. (c) The NiO_y_ layer consist of NiO and Ni_2_O_3_. The 2p_3/2_ binding energies of the NiO and Ni_2_O_3_ phases are 854.8 eV, 857.0 eV, respectively [36].

**Supplementary Figure S1.** (a) X-ray photoelectron spectroscopy for sputtered NbO_x_ layer, (b) MBE deposited NbO_2_ layer [17] and (c) sputtered NiO_y_ layer.

**Supplementary Figure S2.** I-V Characteristics for films which were deposited by sputter and MBE.

To activate E-IMT characteristics of NbO_x_, soft-breakdown (electroforming) process is needed for sputtered NbO_x_ device. It is expected that result of crystallization to form NbO_2_ among amorphous NbO_x_ matrix according to previous research. However, MBE NbO_2_ film is not needed electroforming because the film was already crystallized. Because there is no longer a need for electroforming, the E-IMT process in MBE-deposited NbO_2_ film can be precisely analyzed without the complicating effects of electroforming.


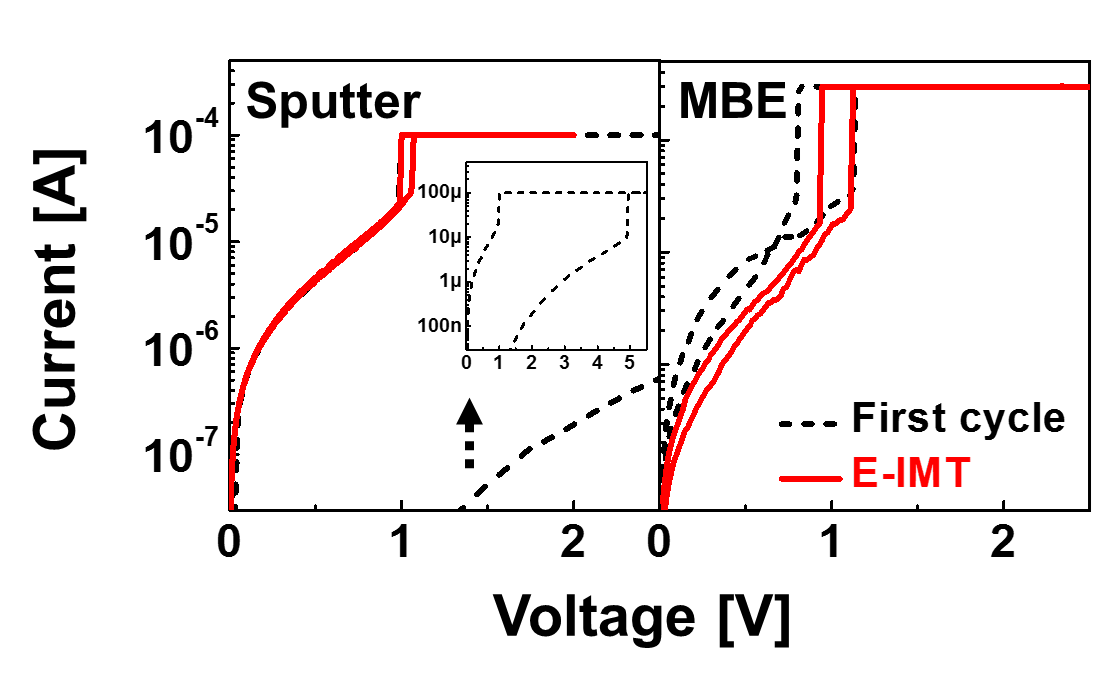


**Supplementary Figure S2.** I-V Characteristics for films which were deposited by sputter and MBE. MBE deposited NbO_2_ film has electroforming-free characteristics thanks to its pristine poly-crystalline state.

**Supplementary Figure S3.** Schematic diagram of electroforming and E-IMT process of sputter- and MBE-deposited films.


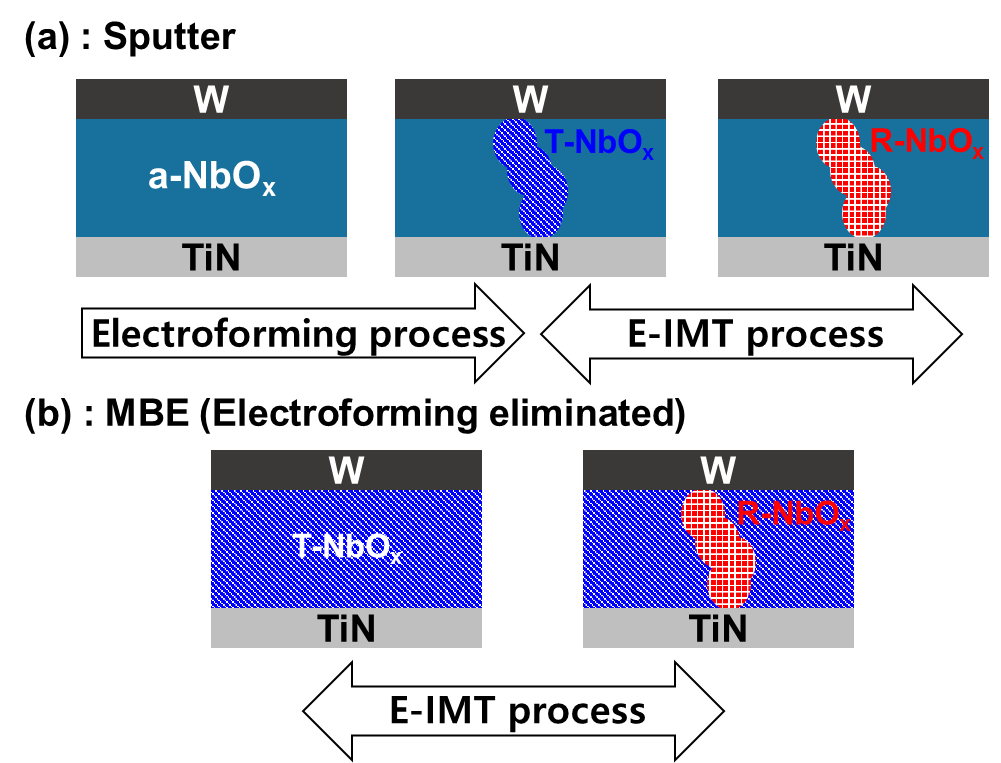


**Supplementary Figure S3.** (a) The schematic diagram of transition mechanism of sputter deposited NbO_x_ film, the electroforming process is needed to crystallize tetragonal NbO_2_ (T-NbO_2_) region. (b) The schematic diagram of transition mechanism of MBE deposited NbO_2.0_ film. Electroforming process is not needed because MBE deposited NbO_2.0_ film was already crystallized T-NbO_2_ in a whole region.

**Supplementary Figure S4.** TEM image of device structure of MBE deposited NbO_2_ film.

**
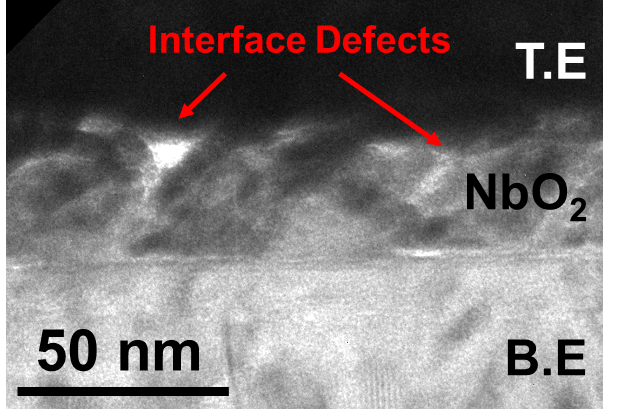
**

**Supplementary Figure S4.** TEM image of device structure of MBE deposited NbO_2_ film. This figure is different region from Fig. 1-(a). Many interface defects such as grain-boundary (G.B), point defects or dislocation are observed between electrode and MBE deposited NbO_2_ region (white region between electrode and NbO_2_)

**Supplementary Figure S5.** Schottky barrier schematic diagram with Richardson relation analysis of W/NbO_x_/W and W/NiO_y_/NbO_x_/NiO_y_/W structure


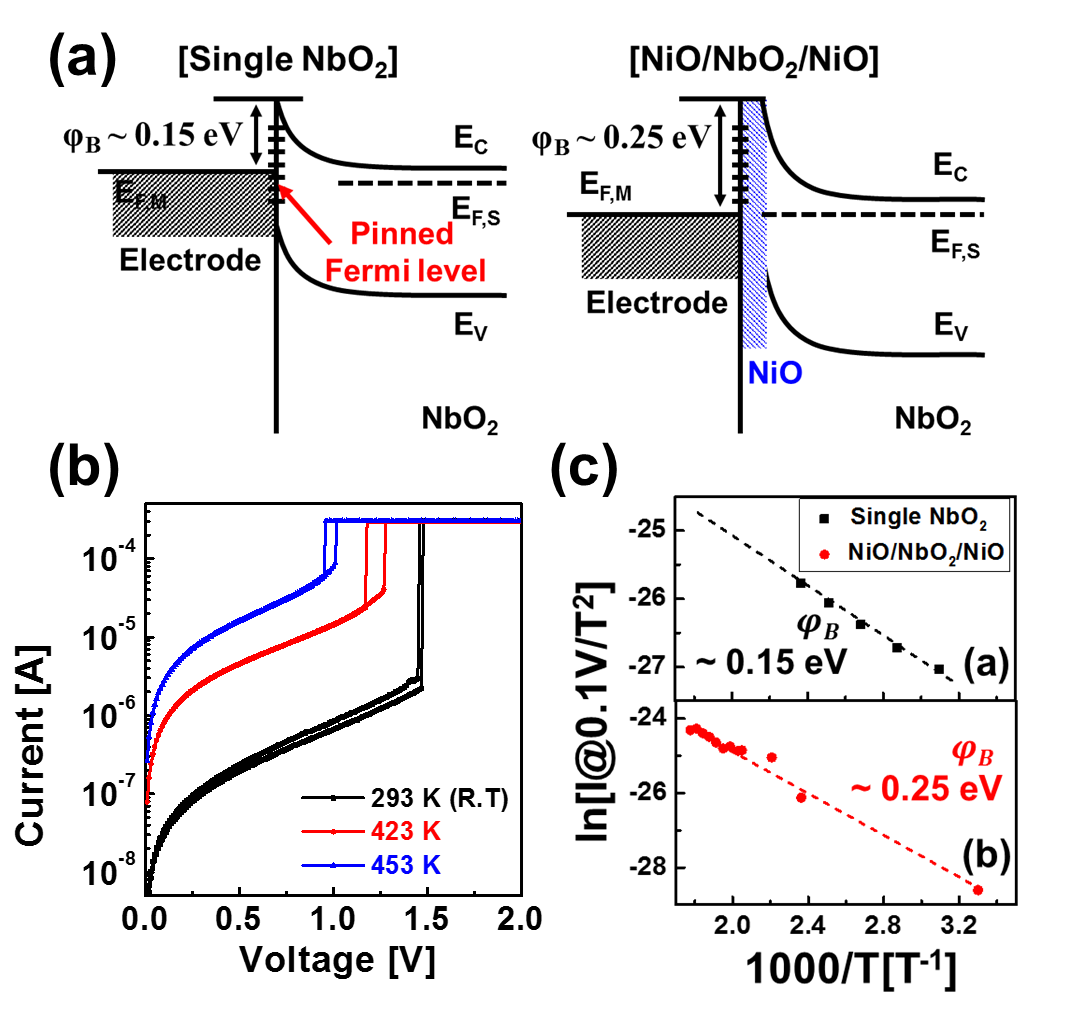


**Supplementary Figure S5.** (a) Schematic diagram of M-S contact energy diagram with schottky barrier degradation in single NbO_x_ device due to defects and recovered schottky barrier in NiO_y_/NbO_x_/NiO_y_ device. (b) The temperature dependency versus I-V characteristic of NiO_y_/NbO_x_/NiO_y_ device and (c) Schottky barrier Richardson plot of both devices.

**Supplementary Figure S6.**

**
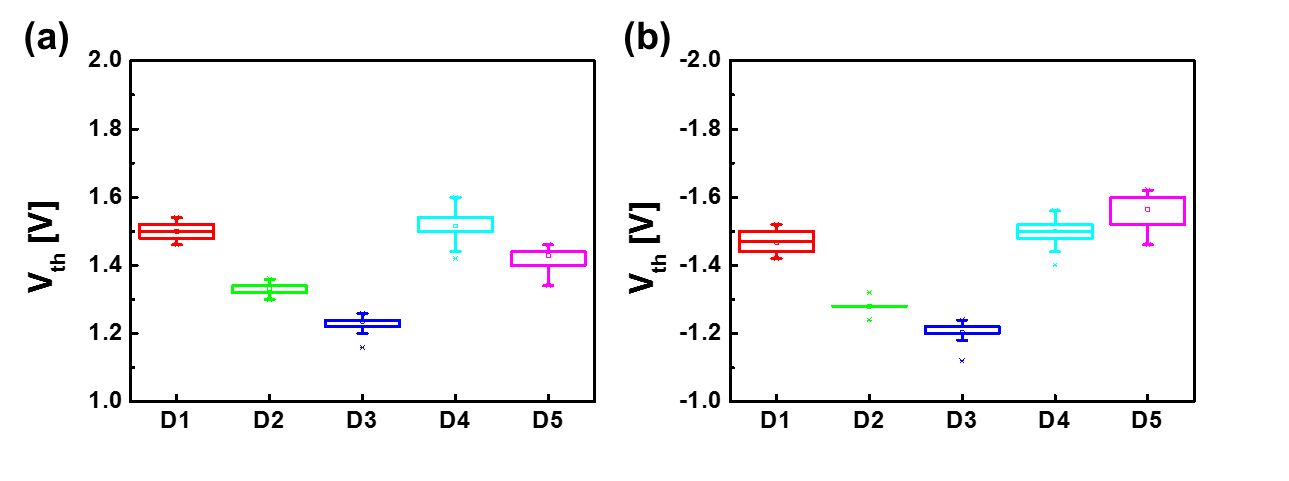
**

**Supplementary Figure S6.** (a-b) Threshold voltage (Vth) distribution of 5 W/NiO_y_/NbO_x_/NiO_y_/W devices during 50 I-V sweeps on both positive and negative biasing polarities.

**Supplementary Figure S7.**


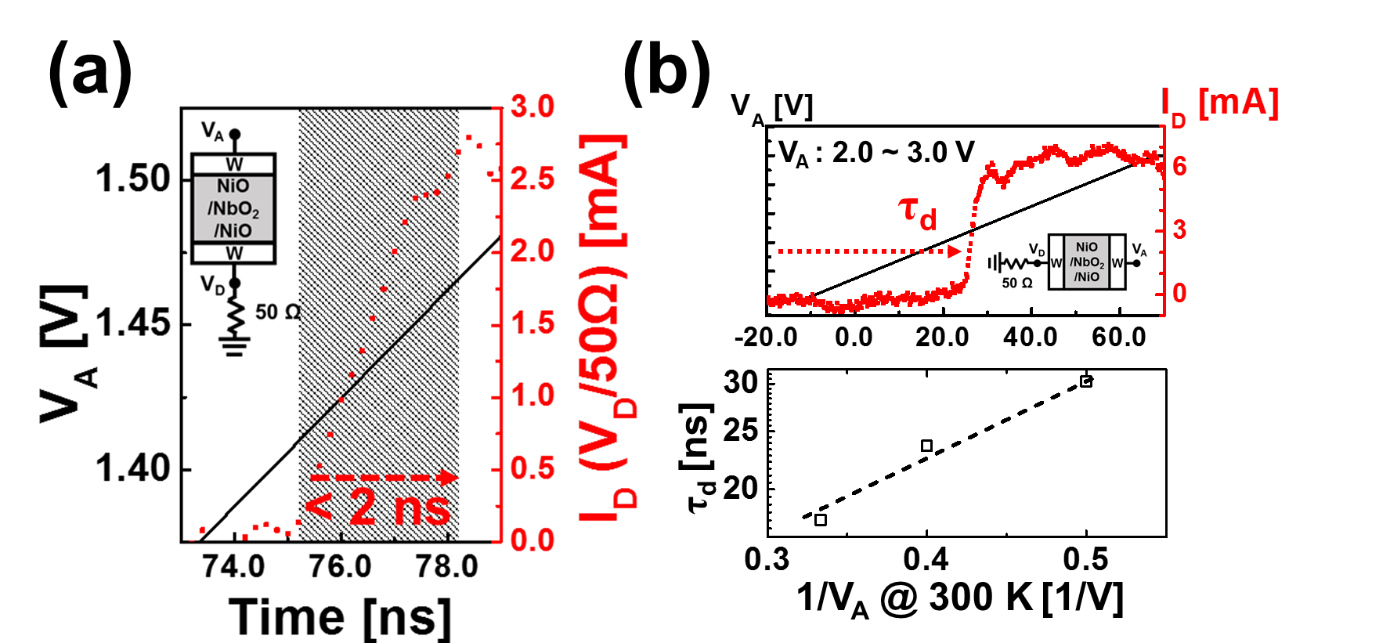


**Supplementary Figure S7.** (a) Transition speed and (b) delay time of NiO_y_/NbO_x_/NiO_y_ device.
